# SOX9 is a proliferation and stem cell factor in hepatocellular carcinoma and possess widespread prognostic significance in different cancer types

**DOI:** 10.1371/journal.pone.0187814

**Published:** 2017-11-09

**Authors:** Georg Richtig, Ariane Aigelsreiter, Daniela Schwarzenbacher, Anna Lena Ress, Jan Basri Adiprasito, Verena Stiegelbauer, Gerald Hoefler, Silvia Schauer, Tobias Kiesslich, Peter Kornprat, Thomas Winder, Florian Eisner, Armin Gerger, Herbert Stoeger, Rudolf Stauber, Carolin Lackner, Martin Pichler

**Affiliations:** 1 Institute of Experimental and Clinical Pharmacology, Medical University of Graz, Graz, Austria; 2 Institute of Pathology, Medical University of Graz, Graz, Austria; 3 Department of Internal Medicine, Division of Oncology, Medical University of Graz, Graz, Austria; 4 Research Unit for Non-Coding RNAs and Genome Editing in Cancer, Medical University of Graz, Graz, Austria; 5 Department of Internal Medicine, Paracelsus Medical University, Salzburger Landeskliniken, Salzburg, Austria; 6 Institute of Physiology and Pathophysiology, Laboratory for Tumour Biology and Experimental Therapies (TREAT), Paracelsus Medical University, Salzburg, Austria; 7 Department of Surgery, Division of Visceral Surgery, Medical University of Graz, Graz, Austria; 8 Department of Oncology, University Hospital Zurich, Zurich, Switzerland; 9 Department of Internal Medicine, Division of Gastroenterology and Hepatology, Medical University of Graz, Graz, Austria; 10 Department of Experimental Therapeutics, The University of Texas MD Anderson Cancer Center, Houston, TX, United States of America; University of North Carolina at Chapel Hill School of Medicine, UNITED STATES

## Abstract

SOX9 has been previously shown to be involved in hepatocellular carcinoma (HCC) and other types of cancer. However, prognostic studies so far involved rather small cohorts or lack external validation and experimental data. In this study, we firstly determined the histological expression pattern of SOX9 in human HCC by immunohistochemistry (n = 84) and evaluated its prognostic value. External cohorts of publicly available datasets were used to validate its prognostic relevance in HCC (n = 359) and other types of cancer including breast (n = 3951), ovarian (n = 1306), lung (n = 1926) and gastric cancer (n = 876). Functional SOX9 knock-down studies using siRNA and cancer stem cell models were generated in a panel of liver and breast cancer cell lines. High level of SOX9 was associated with poor survival even after adjustment for other prognostic factors in multivariate analysis (HR = 2.103, 95%CI = 1.064 to 4.156, *p* = 0.021). SOX9 prevailed a poor prognostic factor in all cancer validation cohorts (p<0.05). Reduced SOX9 expression by siRNA decreased the growth of liver cancer cells (*p*<0.05). SOX9 expression was associated with stem cell features in all tested cell lines (*p*<0.05). In conclusion, this study demonstrated in a large number of patients from multiple cohorts that high levels of SOX9 are a consistent negative prognostic factor.

## Introduction

Hepatocellular carcinoma (HCC) is still a major global problem in health care systems regarding that HCC is the third leading cause of cancer-related deaths worldwide.[1] Many risk factors for the development of HCC have been well characterized including hepatitis B and C virus infection, metabolic syndrome, cirrhosis and alcohol abuse.[2] Genomic analysis revealed several mutations in HCC including mutations in the tumour suppressor gene *p53*, in the telomerase reverse transcriptase gene *TERT* (60% of all cases), and in *CTNNB1* (~30% of HCC cases).[3,4] Other common mutations affect RAS signalling, chromatin remodelling (*ARID1A*) and mammalian target of rapamycin (mTOR) signalling.[4] However, only a few targets are nowadays accessible for approved cancer drugs.[5] Therefore, tumour progression and/or local recurrence of HCC are still a significant problem for long term survival in these patients.[6,7] The Barcelona Clinic Liver Cancer classification is an evidence based algorithm which links therapeutic allocation policies to tumour stages.[8] According to this classification there roughly exist two major treatment strategies: on the one hand radical surgical therapy (including liver transplantation) and percutaneous ablation, both with the goal of curative intention. On the other hand, palliative treatment measures including sorafenib, regorafenib (multi-kinase inhibitors) and chemoembolization.[9,10] The 5-year survival rate within the first group of patients is up to 70% and the median overall survival (OS) is more than 60 months. However, within five years the majority of patients (~70%) develop metastatic disease or *de novo* HCC.[11]

Therefore, there is a great medical need for novel prognostic factors to adequately identify and classify HCC patients who are at higher risk for shortened survival. Further, this might be helpful in better patient stratification and thus monitoring for disease progression.[12].

The transcription protein SRY-box 9 (SOX9) is a member of the high-mobility-group box class DNA-binding proteins and centrally involved in human development processes.[13] It keeps the target cell within an undifferentiated state during development [14] and within this purpose several important pathways like Notch, TGFβ/SMAD and Wnt-β-catenin are additionally involved.[15,16] However, it could also be demonstrated that dys-regulation of SOX9 expression leads to tumourigenesis in several tissue subtypes and high expression of SOX9 enhanced the ability of cancer cells to metastasize.[17–19]. The aim of our study was therefore to examine the significance of SOX9 in prognosis of HCC and across other types of cancer.

## Materials and methods

### Study cohorts

Our retrospective HCC screening cohort consists of eighty-two patients with primary, histologically confirmed HCC who underwent a curative liver resection at the Medical University of Graz, Austria. Tissues used in the study were retrieved from the Institute of Pathology, Medical University of Graz, Austria. We included consecutive patients diagnosed with HCC between January 1988 and December 2009, where sufficient amount of tissue for immunohistochemical analysis was available. None of the patients received neoadjuvant therapy or preoperative local treatment and all underwent tumour resection. Postoperative surveillance was performed at the Division of Gastroenterology or Division of Oncology, Medical University of Graz, Austria, including routine clinical and laboratory examinations every third month, computed tomography scans of the abdomen, and radiographs of the chest every third month. After five years, the examination interval was extended to 12 months. The 7th edition of the American Joint Committee on Cancer (AJCC) / International Union Against Cancer (UICC) TNM system was used to classify the patients.[20] This study was designed to comply with the Declaration of Helsinki. All study participants provided written informed consent if recommended by the guidelines of the local ethics committee and the study was approved by the ethics committee (Approval ID: 24–557 ex 11/12) at the Medical University of Graz (IRB00002556), Graz, Austria.

The second HCC validation cohort consisted of 359 cases of the cancer genome atlas (TCGA) and data have been retrieved by the publicly available http://www.oncolnc.org/.[21] For testing the possible impact of SOX9 gene expression on clinical outcome in different types of cancer, we analysed clinical outcome in breast, ovarian, lung and gastric cancer patients using Kaplan-Meier Plotter (www.kmplot.com). This online available software tool combines Affymetrix gene expression data from multiple annotated cancer studies into a single database which can be then queried for associations of patient outcome with the expression of individual genes.[22] An optimal cut-off value was calculated by the software to dichotomize the cohort according to the SOX9 expression values and significant differences with regard to endpoint overall survival were tested by the log-rank test. Hazard ratio and 95% confidence interval was calculated and a p-value of <0.05 was considered as statistically significant.

### Immunohistochemistry

For SOX9 immunohistochemistry, deparaffinised tissue sections were pre-treated in sodium citrate buffer (0.01 mM) for 40 min in a pressure cooker, followed by 20 min at room temperature. Slides were briefly washed with PBS. Blocking was done with 3% H_2_O_2_ in 90% Methanol for 20 min, followed by brief washing with PBS. Additionally, the Lab Vision™ Ultra V Block (Thermo Fisher, Waltham, MA) was applied and incubated at room temperature for 5 min followed by brief washing with PBS. Slides were incubated overnight at 4°C with polyclonal rabbit antibody to human SOX9 (ab76997, Abcam, Cambridge, MA) diluted 1:100 in DAKO Antibody Diluent S2022 (DAKO, Glostrup, Denmark), followed by another brief washing step with PBS. Detection of binding of SOX9 antibodies was performed with Lab Vision™ UltraVision™ LP Detection System (Thermo Fisher) and with Chromogene Substrate AEC (Dako) for 6 min. After immunostaining, sections were counterstained with hematoxylin and mounted with Aquatex (Merck, Darmstadt, Germany). Primary antibodies were omitted and replaced by diluent for negative control.

The frequency of SOX9-positive cells was estimated by 2 independent, experienced pathologists on a semi-quantitative scoring system, where immunoreactivity was categorized as 0%, 1–10%, 11–50%, and 51–100% positive tumour cells. Staining was assessed by counting the percentage of positive cells per 10 high-power fields (HPF) in the area of the cancerous tissue.

### Cell culture and tumour spheres

The human HCC cell lines HepG2 and Hep3B and the human breast cancer cell lines MCF7, BT474 and SUM159 (ATCC) were used and their origin was proven by DNA identity STR-analysis at the Cell bank of the Core Facility of the Medical University of Graz, Austria (Kit: Promega, PowerPlex 16HS System; Cat.No. DC2101). The HCC cell lines HepG2 and Hep3B as well as the luminal A breast cancer cell lines MCF-7 and BT474 were purchased from the American Type Culture Collection (ATCC) and the basal-like cell line SUM159 was obtained from Asterand (Detroid, MI). HepG2 and Hep3B cells were maintained in Minimum Essential Medium with L-Glutamine (Thermo Fisher Scientific, Waltham, MA USA), 10% fetal bovine serum gold (FBS; Biochrom, Cambridge, UK) and 1% penicillin/streptomycin (Gibco, Darmstadt, Germany; for all used cell lines: Penicillin: 10000 Units/ml, Streptomycin: 10.000 μg/ml, Gibco). MCF-7 cells were grown in MEM with Earle's salts containing 2 mmol/l L-glutamine (PAA, Pasching, Austria), 1% sodium pyruvate (PAA), 1% penicillin/streptomycin (Gibco) and 10% FBS gold (Biochrom). SUM159 cells were maintained in Ham`s F12 containing 2 mmol/l L-glutamine (PAA), 2 mmol/l HEPES buffer (Gibco), 5 μg/ml insulin actrapid (Novo Nordisk, Vienna, Austria), 1 μg/ml hydrocortisone (Sigma-Aldrich, Vienna, Austria) and 5% FBS gold (Biochrom). BT474 were cultured in RPMI 1640 (with L-Glutamine, Gibco), 20% FBS gold (Biochrom), 1% penicillin/streptomycin (Gibco) and 10 μg/ml insulin Actrapid (Novo Nordisk). Cells were incubated in a 5% CO_2_ humidified atmosphere at 37°C.

To measure differences in parental adherent growing cells and tumour spheres, which are considered to be enriched of stem/progenitor cancer cells, we established a spheroid growth model as previously described.[23] In detail, the adherent growing cell lines were dissociated into single cells using trypsin/EDTA and seeded in ultra-low attachment flasks (Corning) using in serum-free McCoy5A medium (SFM). SFM was supplemented with 1xB27 supplement (GIBCO), 20 ng/ml human epidermal growth factor EGF (Peprotech, Rocky Hill, NJ, USA), 20 ng/ml human basic fibroblast growth factor FGF (Peprotech), 20 IU/ml Heparin (Baxter, Vienna, Austria), 4 IU/l insulin actrapid (Novo Nordisk, Vienna, Austria) and 1% antibiotic/antimycotic solution (Sigma-Aldrich, Vienna, Austria). Self-renewing capacity of spheres was shown by dissociating the spheres into single cells which were reseeded to yield the next generation of tumour spheres. Spheres were maintained for several passages. Splitting and dissociation of tumour spheres was done using TrypLE Select (GIBCO). RNA was extracted by TRIzol and SOX9 levels were compared between adherent and spheroid growing cells by qRT-PCR referencing to GAPDH and B2M as reference genes.

### SiRNA transfection and WST-1 assay

To test whether loss of function of SOX9 expression influence cellular growth rates of HepG2 cells, we performed SOX9 knock-down experiments. HepG2 cells were transiently transfected with short interfering RNA (siRNA) to knock-down SOX9 (SOX9; 20 nM, Hs_SOX9_3 FlexiTube siRNA, cat. No. SI00007609, Qiagen) using fast forward transfection procedure according to HiPerFect Transfection Reagent (Qiagen) protocol. As a reference, the AllStars Negative Control (20nM, Qiagen) was used.

After transfection, we measured the cellular growth rate at every 24 hours over a period of time of 96 hours by applying the WST-1 proliferation assay (Roche Applied Science, Mannheim, Germany). In detail, after standard trypsinisation, 5000 HCC cells per well were seeded in a 96-well culture plate. After transfection with a siRNA against SOX9 or respective control (AllStars Negative Control, Qiagen), cells were incubated in 100 μl of normal growth medium for 96 h and the WST-1 proliferation reagent (Roche Applied Science) was added every 24 hours according to manufacturer’s recommendations. At each time point, after 3 hours WST-1 incubation colorimetric changes were measured using a SpectraMax Plus (Molecular Devices) at a wavelength of 450 nm with a reference wavelength at 620 nm.

### Quantitative RT PCR (qRT-PCR)

For detection of SOX9 expression in transiently transfected HepG2 cells after siRNA treatment and to compare expression values of SOX9 in adherent growing cells and tumour spheres, total RNA was isolated using a standard TRIzol protocol according to manufacturer’s instructions. For detection of mRNA expression levels after stable transfection experiments, 1 μg of total RNA was reverse transcribed by using QuantiTect Reverse Transcription Kit (Qiagen, Hilden, Germany) according to manufacturer’s protocol. Quantitative RT-PCR was carried out in technical duplicates of biological triplicates using commercially available primers specific for SOX9 (Hs_SOX9_1_SG_SG QuantiTect primer assay, Qiagen), Nanog (fw: 5`-GTC TTC TGC TGA GAT GCC TCA CA-3`; rev: 5`-CTT CTG CGT CAC ACC ATT GCT AT-3`) and Oct4 (fw: 5`-CCC ACA CTG CAG CAG ATC A-3`; rev: 5`-ACC ACA CTC GGA CCA CAT CC-3`). Quantitative RT-PCR was done on a LightCycler® 480 Real-Time PCR System (Roche Diagnostics, Mannheim, Germany) using QuantiTect SYBR Green PCR Kit (Qiagen) according to manufacturer’s protocol. The arithmetic mean of the reference genes GAPDH and B2M was used for normalization and relative gene expression levels were calculated using a standard 2^-ΔΔCT^ method.

### Statistical analysis of clinicopathological parameters

All statistical analyses were performed using SPSS version 17.0 software (SPSS, Chicago, IL) or MedCalc Statistical Software version 16.8.4 (MedCalc Software bvba, Ostend, Belgium; https://www.medcalc.org; 2016). Fisher’s exact test, χ^2^ test, and the Mann-Whitney procedure were used where appropriate to analyse protein expression in relation to each clinico-pathological parameter. 3-year overall survival of patients was calculated using the Kaplan-Meier method. Backward stepwise multivariate Cox proportion analysis was performed to determine the influence of SOX9 expression, T stage, tumour grade, patient age, presence of cirrhosis and sex on overall survival. Hazard ratios (HRs) estimated from Cox models were reported as relative risks with corresponding 95% confidence intervals (CIs). A P value of <0.05 was considered significant.

## Results

### Patients' characteristics and histopathology

Clinico-pathological patient characteristics including sex, age, tumour stage, tumour grade, growth pattern and presence of liver cirrhosis can be found in Table 1. The majority of HCCs showed a trabecular tumour growth (37/82, 45.1%), followed by trabecular-tubular (24/82, 29.3%) and trabecular-solide (9/82, 11.0%) growth pattern, respectively. Tumour grades were G1 in 22 cases (26.8%), G2 in 49 cases (59.8%) and G3 in 10 (12.2%). Overall, there were more HCCs in male patients (63 cases, 76.8%), in cirrhotic livers (52 cases, 63.4%), with tumour stage III (33 cases, 40.2%) and with trabecular growth pattern (37 cases, 45.1%).

**Table 1:** Clinicopathological characteristics of HCC patients in this study.

Clinicopathological parameter	No. of patients (n = 82)(%)
**Sex**	
Male	63/82 (76.8%)
Female	19/82 (23.2%)
**Age at diagnosis**	
≤65	41/82 (50.0%)
>65	41/82 (50.0%)
**Tumour stage**	
T1	13/82 (15.9%)
T2	24/82 (29.3%)
T3	33/82 (40.2%)
T4	8/82 (9.8%)
Missing	4/82 (4.9%)
**Tumour grade**	
G1	22/82 (26.8%)
G2	49/82 (59.8%)
G3	10/82 (12.2%)
Missing	1/82 (1.2%)
**Growth pattern**	
Trabecular	37/82 (45.1%)
Trabecular-tubular	24/82 (29.3%)
Trabecular-solide	9/82 (11.0%)
Tubular	2/82 (2.4%)
Fibro, trabecular-tubular-solide	5/82 (6.1%)
Fibro-lamellar	3/82 (3.7%)
Solide	2/82 (2.4%)
**Cirrhosis**	
Yes	52/80 (63.4%)
No	28/80 (34.1%)
**SOX9 expression**	
Positive	28/82 (34.1%)
Negative	54/82 (65.9%)

### Histological and immunohistochemical analysis

**Fig 1:**
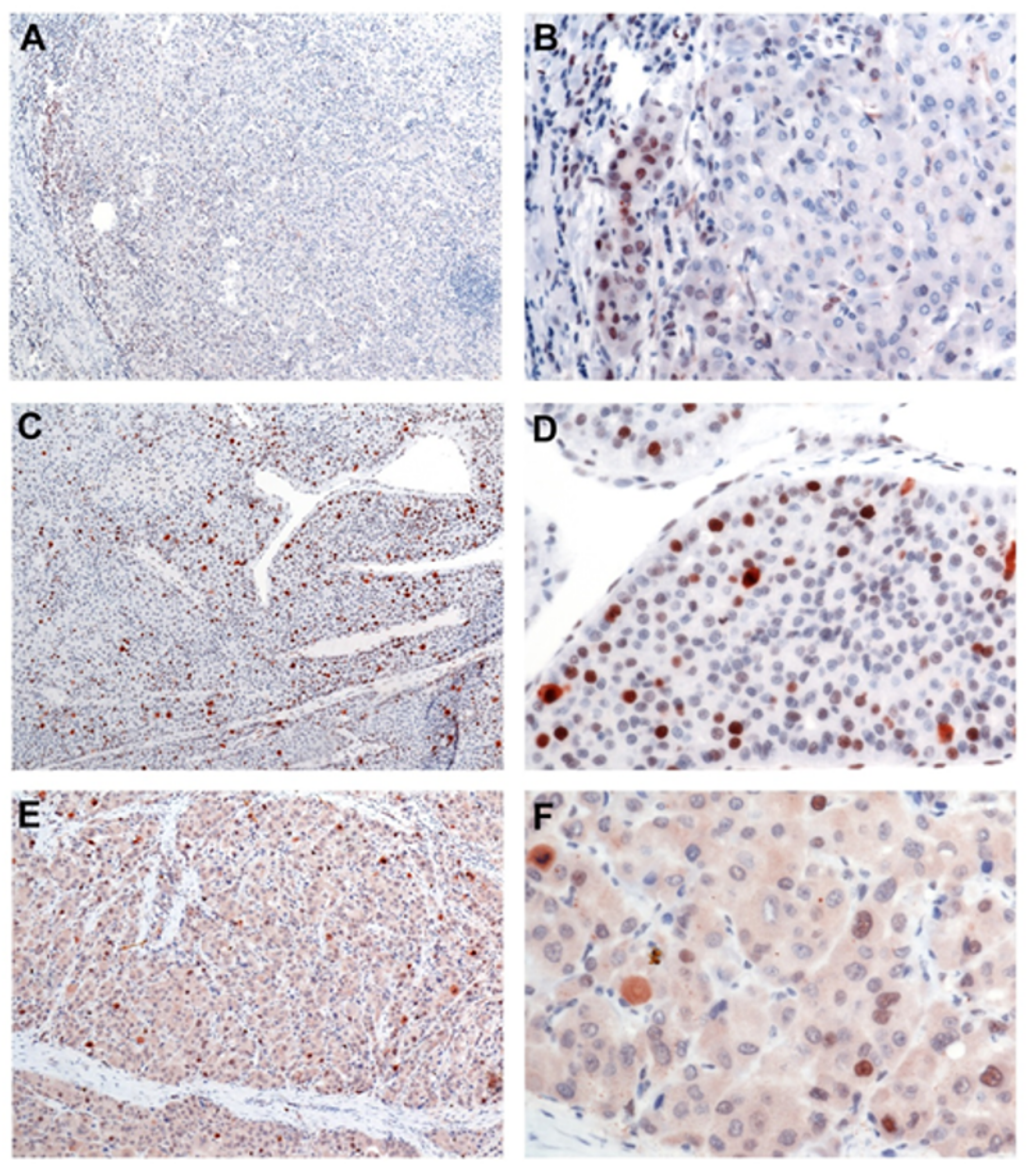
Immunohistochemical expression of SOX9 in hepatocellular carcinomas. **A and B**: Hepatocellular carcinoma mostly negative for SOX9 expression. SOX9 positivity only in single cells (A, overview, 4x magnification). Pronounced SOX9 expression at the invasion front oft the tumor (B, detail, 20x magnification). **C and D**: Hepatocellular carcinoma with SOX9 positivity in 10–20% of tumor cells (C, overview, 4x magnification). D: Detail of HCC with trabecular pattern, positivity more pronounced at the external tumor cell plates (detail of HCC, 20x magnification). **E and F**: Hepatocelluar carcinoma with SOX9 expression in 5% of tumor cells (E, overview, 4x magnification). Beside SOX9 positive nuclei some cells with mitotic figures are highlighted. F: Detail of HCC with few positive nuclei and one mitotic figure in the upper left corner of the picture (40x magnification).

The cut-off value of positivity for SOX9 staining was set to 5% positive tumour cells/per 10 HPF. Using this definition, we found 28 of 82 (34.1%) cases positive for SOX9 expression and 54 (65.9%) cases negative for SOX9 expression. In general, SOX9 was located in the nucleus and mainly displayed a strong staining pattern. Majority of SOX9 positive HCC cases were negative for SOX9 expression in large portions of tumour tissue and SOX9 positive staining’s were found only in single cells (Fig 1A). At the border of the tumour at the invasion front, a pronounced SOX9 expression was seen in a subset of cases (Fig 1B). Hepatocellular carcinoma with SOX9 positivity in a majority of tumour cells (more than 50% of tumour cells) displayed a nearly uniform expression throughout the tumour (Fig 1C). However, in HCC with trabecular pattern, a more pronounced positivity at the tumour cell plates adjacent to sinusoid equivalents (here termed as “external cells”) could be seen in some cases (Fig 1D). Beside SOX9 positivity in the nuclei, SOX9 expression was frequently observed in cells with mitotic figures (Fig 1E and 1F).

### Association of SOX9 expression with clinico-pathological characteristics, its prognostic value and effects of knock-down

To evaluate whether SOX9 protein expression was associated with either clinico-pathological parameters or outcome of patients with HCC, we correlated immunohistochemical SOX9 staining results with tumour stage, age, tumour grade, sex and presence of inclusion bodies, Mallory-denk bodies (MDB), intrahepatic B cells (IHB) or cirrhosis. SOX9 positivity was only significantly associated with male gender (*p* = 0.013, χ^2^ test), whereas all other tested conditions (age (*p* = 0.641, χ^2^ test), presence of cirrhosis (*p* = 0.225, χ^2^ test), tumour stage (*p* = 0.748, χ^2^ test), tumour grade (*p* = 0.075, χ^2^ test), MDB (*p* = 0.583, χ^2^ test), IHB (*p* = 0.078, χ^2^ test) and inclusion bodies (*p* = 0.583, χ^2^ test)) were not significantly associated with SOX9 positivity(detailed in Table 2).

**Table 2:** Frequency of the clinic-pathological characteristics and expression of SOX9 in study cohort with HCC.

Clinico-pathological parameters	SOX9 positive staining (%)	SOX9 negative staining (%)	P
**Sex (n = 82)**			**.013**^**a**^
Male	26 (31.7%)	37 (45.1%)	
Female	2 (2.4%)	17 (20.7%)	
**Age (n = 82)**			.641^**a**^
≤65	15 (18.3%)	26 (31.7%)	
>65	13 (15.9%)	28 (34.1%)	
**Cirrhosis (n = 80)**			.225^**a**^
Yes	20 (25.0%)	32 (40.0%)	
No	7 (8.8%)	21 (26.3%)	
**Tumour stage (n = 78)**			.748^**a**^
Low tumour stage (T1+T2)	13 (16.7%)	24 (30.8%)	
High tumour stage (T3+T4)	13 (16.7%)	28 (35.9%)	
**Tumour grade (n = 81)**			.075^**a**^
Low grade (Grade 1)	11 (13.6%)	11 (13.6%)	
High grade (Grade 2 & 3)	17 (21.0%)	42 (51.9%)	
**MDB (n = 74)**			.583^**a**^
No MDB	15 (20.3%)	23 (31.1%)	
MDB	12 (16.2%)	24 (32.4%)	
**IHB (n = 74)**			.078^**a**^
No IHB	21 (28.4%)	27 (36.5%)	
IHB	6 (8.1%)	20 (27.0%)	
**Inclusion bodies (n = 74)**			.583^**a**^
No inclusion bodies	15 (20.3%)	23 (31.1%)	
One or both inclusion bodies	12 (16.2%)	24 (32.4%)	
**Grouped (n = 74)**			.339^**a**^
No inclusion	13 (17.6%)	18 (24.3%)	
IHB	2 (2.7%)	5 (6.8%)	
MDB	8 (10.8%)	9 (12.2%)	
IHB and MDB	4 (5.4%)	15 (20.3%)	

^a^χ^2^ test; MDB = Mallory-Denk body; IHB = intracellular hyaline bodies

**Fig 2:**
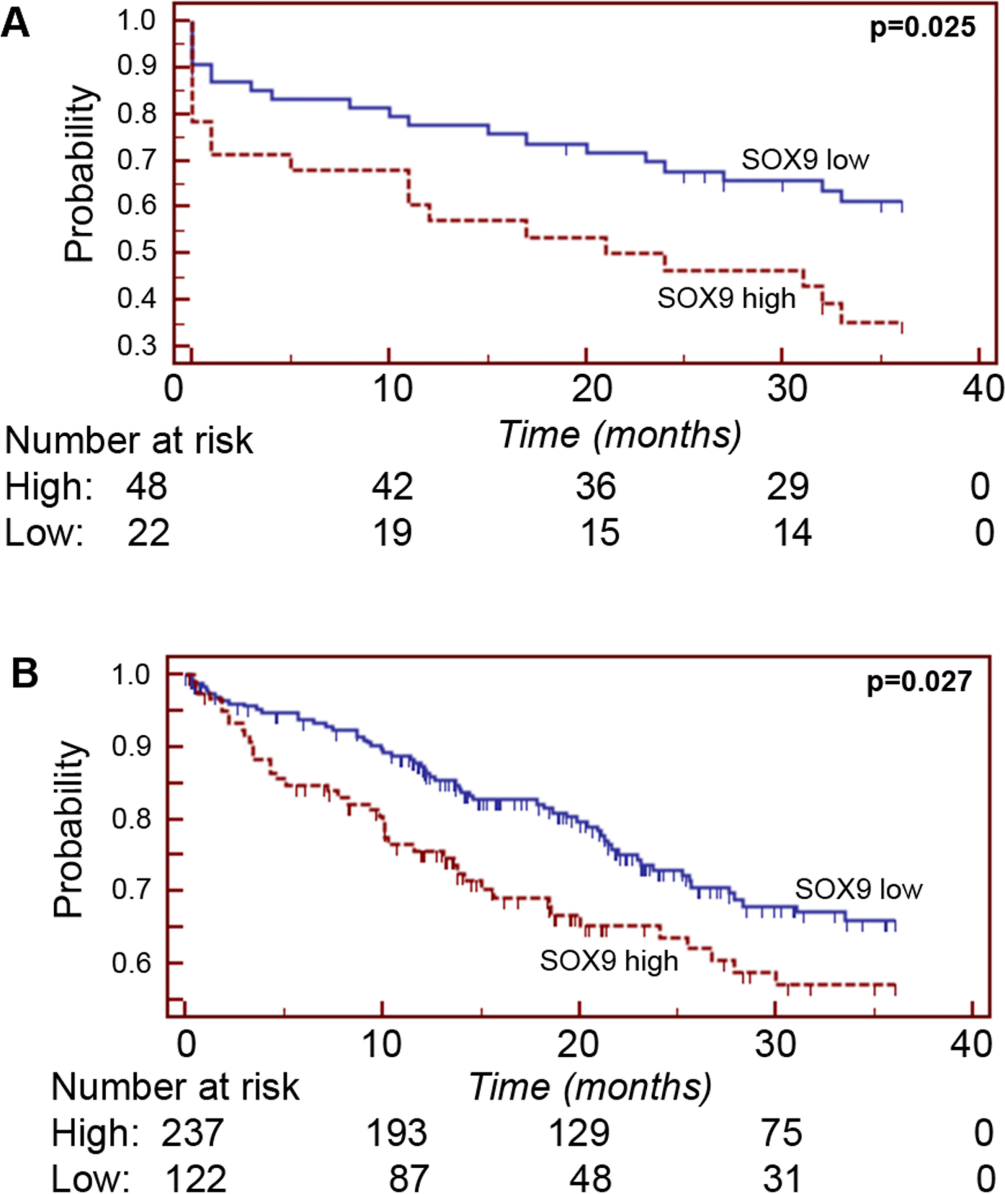
Kaplan-Maier plot for SOX9 expression in HCC. A: Kaplan-Maier plot for 3-year survival in patients with HCC showing immunohistochemical positive SOX9 expression versus negative SOX9 expression (n = 84). B: Kaplan-Maier plots for 3-year survival calculated from a dataset derived from the Cancer Genome Atlas in patients with HCC showing high SOX9 expression versus low SOX9 expression.

Overall, three-year overall survival was observed in 44 (53.7%) of 82 patients. We divided HCC patients according to the cut-off value of 5% into the groups SOX9 positive (n = 28) and SOX9 negative (n = 54), respectively. For the 3-year overall survival, death occurred in 20 patients (37.0%) of 54 in the SOX9 negative group and 18 (64.3%) of 28 died in the SOX9 positive group (*p* = 0.025, log-rank test). Fig 2A shows the Kaplan-Meier-curve for 3-year overall survival and shows further that there was a significant shorter survival in the SOX9 positive group suggesting that SOX9 expression is a factor for poor prognosis. To determine the independent prognostic value of SOX9 positive staining on three year overall survival, a multivariate Cox proportional hazard analysis was performed including sex, age, tumour stage, tumour grade and SOX9 protein expression. SOX9 positive protein expression (HR = 2.10, 95% CI = 1.06 to 4.16, *p* = 0.021) was also identified by multivariate analysis as an independent predictor for 3-year overall survival. In addition, tumour stage turned out as an independent prognostic factor for HCC patients (HR = 2.72, 95% CI = 1.27 to 5.83, *p* = 0.010).

**Fig 3:**
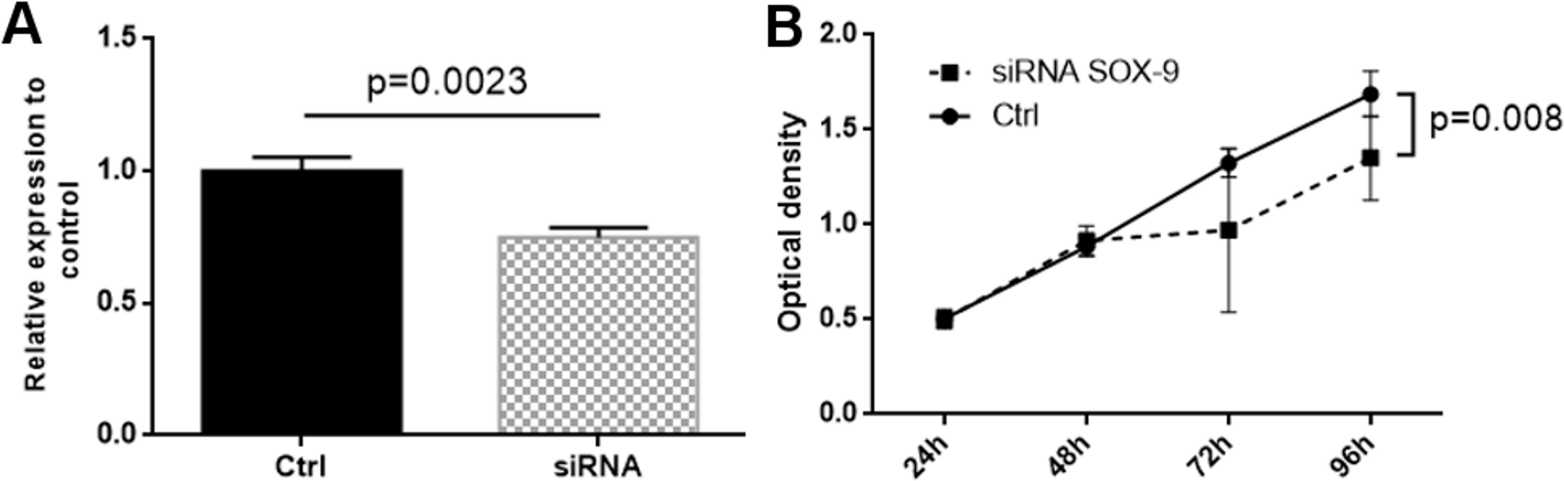
(A) SOX9 knock-down was assessed by expression analysis of mRNA by qRT-PCR after siRNA treatment against SOX9. The dark grey bar shows a significant decrease in SOX9 expression in HepG2 cells. (b) HepG2 cells show reduced cellular growth after SOX9 knock-down after 96 hours follow-up.

To externally validate our findings in an independent cohort, we explored the Cancer Genome Atlas dataset (TCGA data, n = 359 patients with HCC) and used the same grouping proportions (66% of patients defined as low and 34% of patients as high expression) for categorizing patients. As shown in Fig 2B, patients with high SOX9 expression showed again a poor prognosis in this large independent dataset (*p* = 0.027). To further elucidate some possible cellular effects of SOX9 expression in HCC cancer cells, we used short interfering RNAs (siRNAs) to successfully knock-down the expression in HepG2 cells (Fig 3A). After SOX9 knock-down, cellular growth assays indicate slower growth rate of HCC cells with low SOX9 expression levels after 96 hours (*p* = 0.008, Fig 3B).

### Prognostic power of SOX9 in other cancers and its relation to cancer stem cell traits

**Fig 4:**
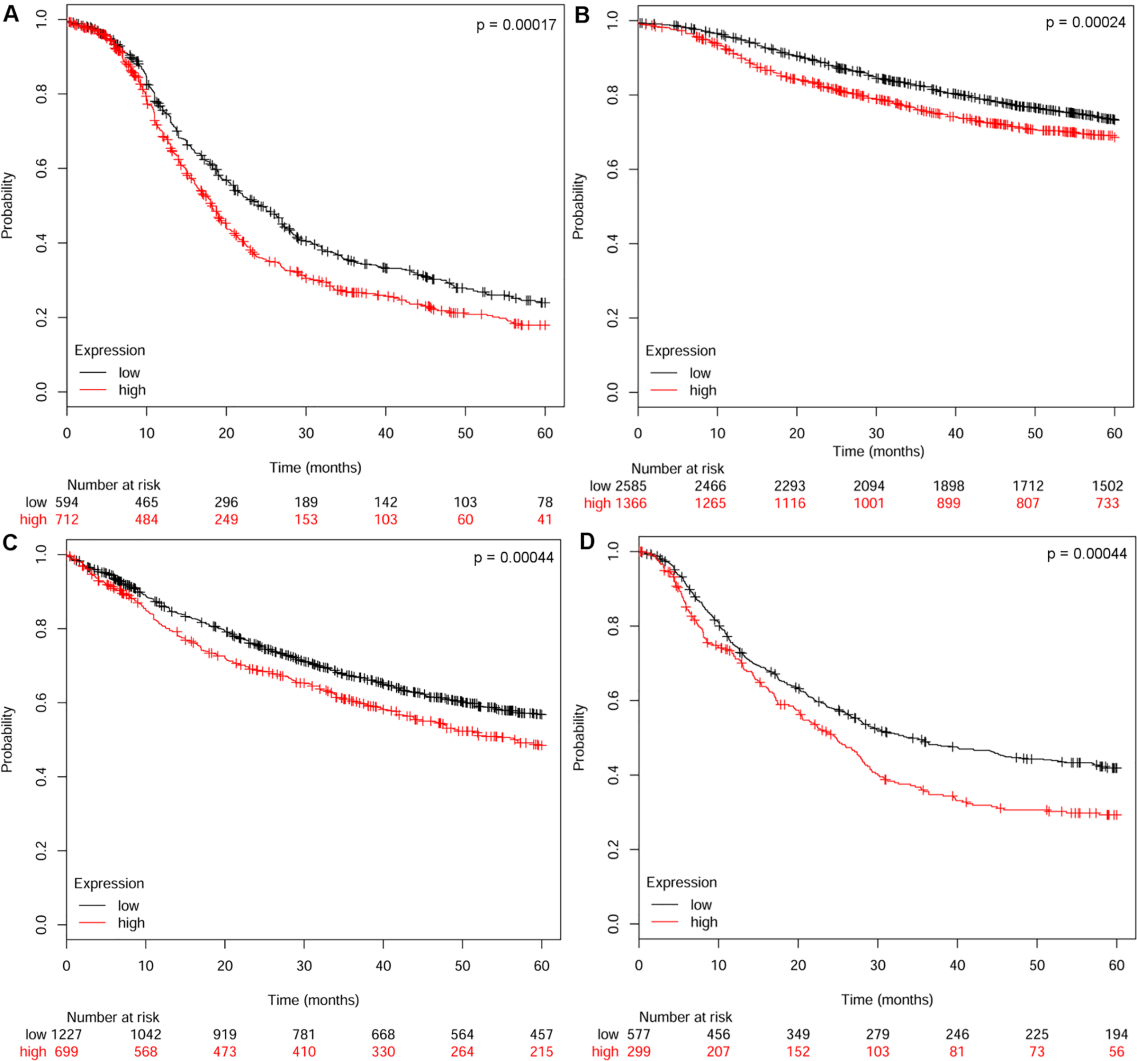
Prognostic significance of SOX9 across several cancer types. (A) 5-year relapse free survival rates in breast cancer patients. (B) 5-year progression free survival in ovarian cancer patients. 5-year survival rates in patients with (C) lung cancer and (D) gastric cancer.

To test for universality of SOX9 expression as prognostic factor in other cancers, we additionally analysed unbiased publicly available datasets of breast (n = 3951), ovarian (n = 1306), lung (n = 1926) and gastric cancer (n = 876). Regardless of the type of cancer, high SOX9 expression levels always and consistently prevailed as strong predictor of poor 5-years relapse free survival (breast, Fig 4A), progression-free survival (ovarian cancer, Fig 4B) and overall survival (lung and gastric cancer, Fig 4C and 4D).

**Fig 5:**
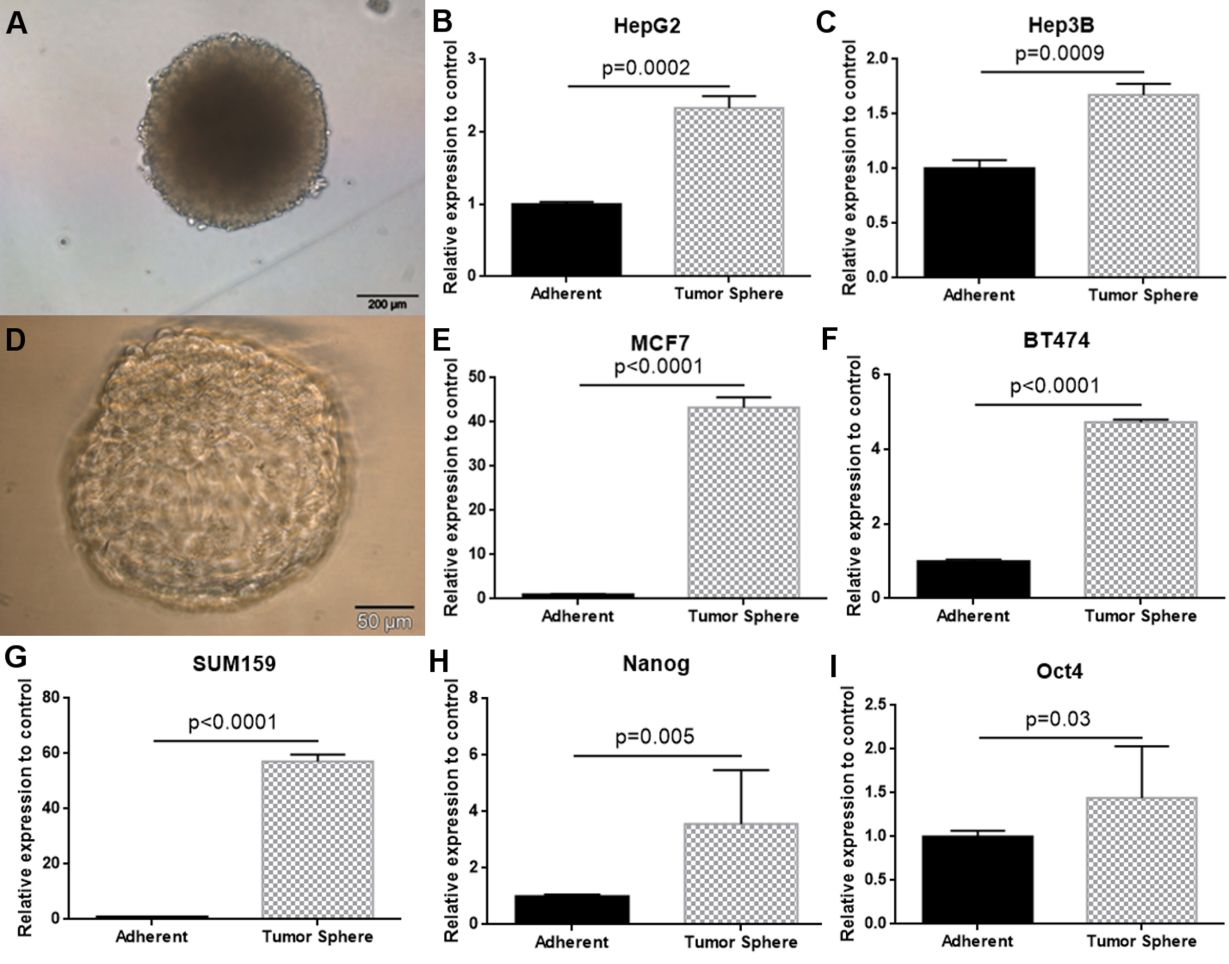
Representative examples of tumour spheres in hepatocellular carcinoma (HepG2)(D) and breast cancer (MCF7)(A) (D) cells. SOX9 expression has been assessed by qRT-PCR in two liver cancer cell lines (HepG2 (B) and Hep3B (C)) and in three breast cancer cell lines (MCF7 (E), BT474 (F) and SUM159 (G)) comparing parental adherent or tumour spheres. In all five tested cell lines, SOX9 was significantly higher expressed in tumour spheres (between 1.6-fold and 57-fold increased) than in parental adherent cells. Expression levels of two cancer stem cell marker (Nanog (H) and Oct4 (I)) have been determined all five cancer cell lines in adherent cells and tumour spheres. Nanog and Oct4 were significantly up-regulated in tumour spheres in comparison to normal adherent tumour cells.

As SOX9 has been previously discussed as a factor involved in stem cell biology, we generated tumour spheres in a panel of liver and breast cancer cell lines (n = 5) under low attachment condition. This *in vitro* model system is commonly used to test stem cell capabilities in cancer cells. We measured significantly higher SOX9 expression levels in all tested tumour spheres compared to their adherent growing parental cell line (Fig 5B, 5C and 5E–5G).

To confirm that our tumour sphere models are enriched with putative cancer stem cells, we measured the established stem cell markers Nanog and Oct4 in the same tumour spheres. Both transcription factors were up-regulated in tumour spheres compared to normal adherent growing tumour cells (Fig 5H and 5I).[24]

## Discussion

SOX9 has been implicated as a cancer stem cell marker with the ability to drive cancer growth and promote cancers metastatic ability[25,26]. Indeed, several groups could show that tissue development and cancer development rely on SOX9. In melanoma SOX9 is necessary to switch melanoma from a proliferative state to an invasive one whereas it regulates about 10% of the genes that define the invasive phenotype gene set[27]. This finding has been confirmed for several other cancer types including HCC[28–30]. In our gene silencing assay we could confirm these results demonstrating that siRNA against SOX9 reduced growth of liver cancer cells. Additionally, SOX9 is significantly enriched in a sphere model system for cancer stemness. In line with our and other’s pre-clinical data, our study could further demonstrate that high SOX-9 expression was associated with shorter overall survival in HCC in two different cohorts, using two different methods comprising overall more than 400 patients. To the best of our knowledge, our study is to date largest one, confirming the findings by other smaller study groups showing that high SOX9 expression was associated with higher tumour stage and lower overall survival independently of the cancer type[27,31].

The positivity for SOX9 expression was independent of the presence of cirrhosis, one of the major risk factors for HCC. On the one hand we could not find any association between SOX9 expression and tumour stage nor age which is in line with others[32]. Compared to Guo *et al*. we could not show any association between tumour grade and SOX9 expression although a trend towards lower expression high grade (G2/3) tumours was seen in our study population[17]. Interestingly, we found a positive correlation between gender and SOX9 expression. Male patients had a significantly higher likelihood to have a tumour positive for SOX9 which is in line to the findings of Xue et al.[26] A recent work by our group could show that HCCs with IHB had significantly shorter overall survival[33]. However, we could not observe any association between MDB, IHB or the combination of both and SOX9 positivity. This suggest that SOX9 might be an independent prognostic factor apart from inclusion bodies in HCC and furthermore is not functionally related to autophagy, though this lacks any experimental data[34]. However, we could further show that SOX9 positivity was associated with poor clinical outcome not only in HCCs but also in lung, breast, gastric and ovarian cancer.

*In vitro* studies could show that SOX9 is also a marker of cancer stem cells in breast cancer and other tumour entities[35,36]. This could also be shown in our study and not surprisingly SOX9 positivity was also associated with shorter relapse free survival in patients with breast cancer. Some pre-clinical work suggested that SOX9 expression contributes to aggressiveness and invasiveness in ovarian cancer, but clinical data are currently sparse[37,38]. Herein, we could show that observed pre-clinical aggressiveness had also a direct impact on prognosis in patients with ovarian cancer. Furthermore our data on the prognosis of lung cancer confirm *in vitro* studies and other clinical studies[31,39,40]. Interestingly, SOX9 was identified as mediator of cisplatin-resistant cancer types but failed to show any impact on overall survival in a study conducted by Sun et al.[41] Furthermore, Zhou *et al*. could show that SOX9 expression was associated with higher tumour staging, tumour stage and higher probability of metastatic spread[42].

In conclusion, our study supports the hypothesis that SOX9 is involved in liver cancer proliferation and HCC stem cell capabilities. Furthermore, SOX9 is a universal prognosis marker across several types of cancer. Therefore, therapeutic strategies targeting SOX9 and it´s up-stream effectors might be helpful in treating SOX9-dependent tumours.
